# Establishing the extent of pesticide contamination in Irish agricultural soils

**DOI:** 10.1016/j.heliyon.2023.e19416

**Published:** 2023-08-23

**Authors:** Mathavan Vickneswaran, James C. Carolan, Matthew Saunders, Blánaid White

**Affiliations:** a School of Chemical Sciences, Dublin City University, Glasnevin, Dublin 9, Ireland; b Department of Biology, Maynooth University, Maynooth, Co. Kildare, Ireland; c Department of Botany, Trinity College Dublin, College Green, Dublin 2, Ireland

**Keywords:** Irish agricultural soil, Monitoring, Pesticide residues, Neonicotinoids, Risk assessment

## Abstract

To establish meaningful and sustainable policy directives for sustainable pesticide use in agriculture, baseline knowledge of pesticide levels in soils is required. To address this, five pesticides and one metabolite widely used in Irish agriculture and five neonicotinoid compounds pesticides were screened from soils from 25 fields. These sites represented a diversity of soil and land use types. Prothioconazole was detected in 16 of the 18 sites where it had been recently applied, with the highest maximum concentration quantified of 46 μg/kg. However, a week after application only four fields had prothioconazole concentrations above the limit of quantification (LOQ). Fluroxypyr was applied in 11 sites but was not detected above LOQ. Glyphosate and AMPA were not detected. Interestingly, neonicotinoids were detected in 96% of all sampling sites, even though they were not reported as recently applied. Excluding neonicotinoids, 60% of sites were found to contain pesticide residues of compounds that were not previously applied, with boscalid and azoxystrobin detected in 15 of the 25 sites sampled. The total number of pesticides detected in Irish soils were significantly negatively correlated with clay fraction, while average pesticide concentrations were significantly positively correlated with log *K*_ow_ values. 17 fields were found to have total pesticide concentrations in excess of 0.5 μg/kg, even when recently applied pesticides were removed from calculations. Theoretical consideration of quantified pesticides determined that azoxystrobin has high leaching risk, while boscalid, which was detected but not applied, has an accumulation risk. This information provides insight into the current level of pesticide contamination in Irish agricultural soil and contributes to the European-level effort to understand potential impacts of pesticide contamination in soil.

## Graphical abstract

**Figure:**
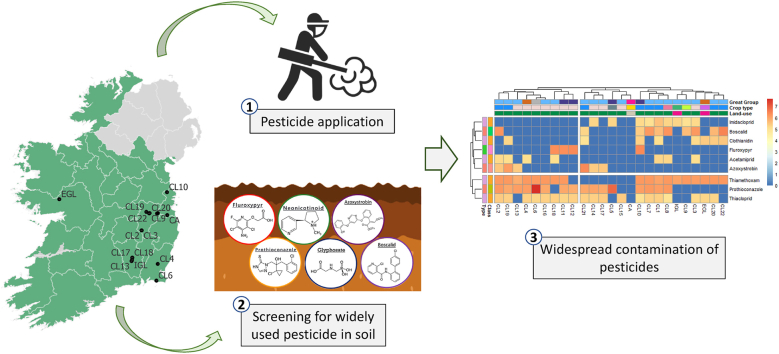

## 1 Introduction

Plant protection products in the form of herbicides, fungicides, and insecticides have become a ubiquitous component of global agricultural management practices, responsible for increased crop productivity and yield and driving agricultural intensification [[1], [2], [3]]. They can be applied throughout all phases of crop cultivation, including as seed treatments prior to planting or after seed germination [4], during crop growth period when pests or diseases are at their highest levels [5,6], or before the shipment of the crop products [[7], [8], [9]]. Agriculture is vital to the economies of many countries, including Ireland, where it directly contributed 4.3% of gross value added or €14.4 billion to the national economy in 2019 [10]. The total land area of Ireland is approximately 6.9 million hectares, of which 4.4 million hectares, or 71%, is used for agriculture [11]. The majority of this farmed area (4.1 million hectares) is used for pasture, hay, and grass silage, with 0.016 million hectares used for rough grazing and 0.29 million hectares devoted to crops, including cereals, potatoes, fruit and horticultural production [11]. To sustain and improve the productivity of the Irish agricultural industry, the utilised agricultural area would reflect the amount of pesticide usage. For instance, between 2016 and 2017, the area of Irish land used for agriculture had increased by 28,310 ha, whereas the total pesticide usage in 2017 was 2861 tonnes, an increase of 323 tonnes from the previous year [[12], [13], [14]]. Despite the increased costs that arise from pesticide use, their use has consistently increased over the past decades. More than 3000 different types of pesticide formulations are used in Europe over the past 55 years, raising serious environmental concerns regarding the intensive release of synthetic chemicals into the environment [2,[15], [16], [17], [18], [19]].

The assumption that pesticides degrade in the environment after achieving their function has been shown to be invalid. It is estimated that about 99.9% of applied pesticides are subjected to off-site transfer [[20], [21], [22]] through numerous simultaneous routes, including volatilisation [23,24], spray drift [25,26], surface run-off [[27], [28], [29]], degradation and leaching [[30], [31], [32]], while only 0.1% of the applied pesticides reach their intended target pest [20]. Additionally, Lechenet et al. (2017) reported that current recommended pesticide application rates exceed what is necessary for efficient crop protection, which could result in higher soil contamination than intended [33]. With increased presence of pesticide in the environment, it increases the potential for humans and other living organisms exposed to pesticide compounds [34]. Furthermore, exposure to pesticides were noted to be hazardous to human behaviour and physiology, with diseases such as hypersensitivity, asthma, cancer, congenital disabilities, reduce birth weight and hormonal disturbances, all established to be linked to hazardous effect of pesticides [[35], [36], [37]].

Pesticide presence in soils can originate from multiple sources; either through aerial spray onto crops where the soil can be exposed to pesticides from drips from the crop [1,38], direct treatment of the soil [39,40], or through seed-coating [[40], [41], [42]]. Once the pesticide is incorporated into the soil, it enters a dynamic environment in which it can display highly variable behaviour, depending on one of the four main processes, adsorption, desorption, degradation or transformation, and leaching [[43], [44], [45]]. However, the efficiency of the process is fundamentally influenced by numerous factors, including the physicochemical properties of the pesticide compound and the soil layer, the climatic condition and soil biotic properties [[45], [46], [47], [48], [49]].

The adsorption and desorption potential of a pesticide residue can be inferred based on their physicochemical properties, namely dissociation constant (pKa), partition coefficient (log *K*_ow_), solubility, adsorption coefficient (*K*_oc_) and vapour pressure [[50], [51], [52], [53]], and based on the soil physicochemical properties such as pH, soil organic matter, and the soil texture [46,50,54]. However, organic matter and clay mineral content principally dictate the levels of pesticide persistence [[55], [56], [57]]. These components are linked [58], and present multiple functional groups onto which pesticide residues can adsorb. In addition, their relatively large and chemically active surface areas increase their chemical affinity for pesticide residue adsorption [59], and although soil organic matter is usually thought to represent a better adsorption matrix compared to clay minerals [[60], [61], [62]], both of these matrices’ presence are highly associated with each other [58]. Although they can exist independently, organic matter in the soil is directly or indirectly governed by clay minerals [58]. Once organic matter interacts with clay minerals, it remains protected from mineralisation, consequently increasing the organic matter content in the soil [58]. Hence, it can be postulated that soil with high clay or high organic matter, or both, has a higher risk of pesticide accumulation. On the other hand, high soil organic matter content would be expected to increase microbial activity [[63], [64], [65], [66]], which increases microbial degradation, the primary degradation route of soil pesticides. Soil microbes can break down pesticides through metabolic processes such as polymerisation, accumulation and conjugation, co-metabolism, and mineralisation [46]. Even though microbial degradation correlates positively with the soil organic matter content [67], the efficiency of the biodegradation process is affected by abiotic properties of the soil, such as aeration, pH moisture, and temperature [50]. Additionally, the uncertainties of climatic conditions, such as rainfall, relative humidity, evaporation, air movement, light and temperature, add to the complexity of predicting pesticide mobility in soil [68].

To date, our knowledge of pesticide occurrence on the island of Ireland derives from studies on groundwater [69], freshwater [[70], [71], [72]], wastewater [73], food and products [[74], [75], [76], [77]], and meta-analyses of the literature [78,79]. Although pesticide contamination of agricultural soils has been assessed for Northern Ireland [2], no equivalent records or studies exist for the Republic of Ireland. To the best of our knowledge, no characterisation of widely used pesticides has been conducted for Irish soils or sites. The principal aim of our study therefore was to provide a baseline overview of pesticide contamination in agricultural soils from 25 sites across the Republic of Ireland and use this information to establish a reference for future monitoring. In total, ten pesticides (five neonicotinoids and five non-neonicotinoids) were selected for analysis. Although neonicotinoids have been banned for outdoor use in Ireland since 2018 [[80], [81], [82]]; their long half-lives (ranging from 3 to 6931 days) and environmental persistence resulted in their inclusion [[83], [84], [85], [86], [87], [88]]. The other pesticides were selected based on their widespread and large-scale use in Irish agriculture [78]. Finally, the glyphosate metabolite aminomethylphosphonic acid (AMPA) was included in the monitoring, given that it can accumulate rapidly and persist in soil environments [89].

Our study evaluates potential correlation between applied pesticide concentration to their chemical properties, the soil physicochemical properties, and the land-use type to provide a broader and more holistic interpretation of the fate of specific pesticides in specific contexts. Additionally, an analysis was carried out of total pesticide concentrations at field level, and an assessment of their potential for leaching from agricultural soils based on the properties of the individual chemicals and specific soil types was completed. It is anticipated that the results of our study will offer insights into pesticide contamination in Irish agricultural soils and form the basis for future pesticide monitoring and management actions.

## 2 Materials and methods

### 2.1 Soil sampling site selection

Sites were selected to represent the variety of soil conditions and agricultural practices across Ireland using the Irish Soil Information System (SIS) [90,91] and the spatially integrated Land use and Soil Inventory for Ireland (LUSSI) [92]. All communications and contact complied with General Data Protection Regulations (GDPR). Soil samples were collected i) from sites within 24 h and one week of pesticide application and ii) from sites with no recent human or agricultural activities or history of pesticide use to serve as non-pesticide controls.

### 2.2 Soil sampling

**Fig. 1:**
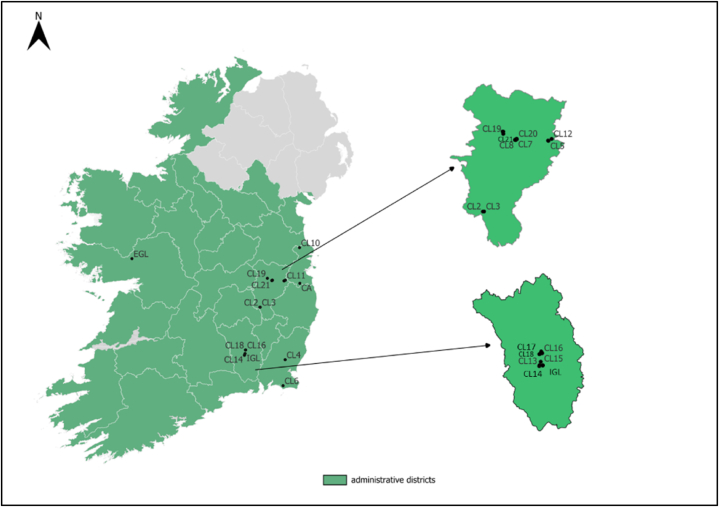
Sampling point map of the research area (Republic of Ireland), callouts are for the counties Kildare (top) and Kilkenny (bottom). The label for each point indicates the field's label: *EGL:* Extensive grassland.; *IGL:* Intensive grassland.; *CL:* Cropland.; *CA:* Commonage Area.

In total, 25 soil samples were obtained from 25 sites (Fig. 1) between April to July 2021. These 25 sites were sampled within 24 h of pesticide application, and were revisited a week after for the collection of samples one week after application. The complete details of the sampled fields and the pesticides applied are highlighted in Table S1. Five soil cores (15 cm depth and 10 cm diameter; topsoil with vegetated tops removed) were collected randomly across each field, focusing on the centre of the field where pesticides were applied. The soil cores were then combined and homogenised to produce a single sample from each site. Approximately one kilogram of the soil mixture was packed in clean plastic Ziplock bags, placed in an icebox (temperature of approximately 4 °C and below), kept in the dark, and transported to the laboratory. All samples were then stored at −20 °C. Prior to further analysis, soils were defrosted, air-dried and sieved using a stainless-steel sieve with a mesh size of 2 mm. Large objects such as roots and stones were removed, and where necessary, a pestle and mortar were used to grind soils further. Sieved soil samples were stored in airtight plastic bags and restored at −20 °C.

### 2.3 Soil physicochemical properties determination

Soil physicochemical properties were determined in triplicate for each site by measuring pH_CaCl2_, texture, organic matter, moisture content, water holding capacity, total nitrogen (TN) and total phosphorus (TP). Clay, silt and sand content were measured using a standard hydrometer (152H ASTM) after dispersion in sodium hexametaphosphate (50 g/L). Fractions were classified as sand, silt and clay following the US Department of Agriculture (USDA) system to distinguish particle size [93,94]. A pH meter was used to measure the pH of air-dried soil samples mixed with 0.01 M CaCl_2_ (at the ratio of 1:5) and left overnight [95]. Moisture content was determined by calculating the difference in weight before and after oven drying fresh soil samples for three days (72 h) or until the soil samples achieved constant weight [96,97]. Water holding capacity was measured using the Haines-funnel system, where the soil samples were saturated with 100 mL of water for 30 min, then the water was drained, and the amount of water retained in the soil was calculated [98]. Oven-dried samples were used to determine total soil organic matter by the loss-on-ignition method. The samples were subjected to 550 °C in a muffle furnace for 6 h and left in the furnace overnight. The total soil organic matter percentage was then calculated from the difference before and after ignition [99]. Cell test kits determined total nitrogen and phosphorus photometrically after digestion. Total nitrogen was determined following Koroleff's digestion method, while total phosphorus was analysed through digestion in sulfuric solution [100,101].

### 2.4 Standards and reagents

All salts and solvents were analytical or LC-MS grade. Acetone, acetonitrile, acetic acid 100%, dichloromethane, petroleum ether, formic acid 98%, sodium hexametaphosphate, anhydrous sodium sulphate, ammonium bicarbonate, ammonium formate, methanol (MeOH), Millipore Millex syringe filters with hydrophilic PTFE membrane (pore size 0.22 μm and 20 mm diameter), PTFE centrifuge tubes (15 mL and 250 mL), and total nitrogen and phosphorus cell test kits were purchased from Merck Life Science (Ireland). Ultrapure water, deionised to the resistance of <18 MΩ x cm was generated using ELGA Purelab Ultra SC MK2 (ELGA, UK). Certified reference analytical standards, >97% purity, of acetamiprid, azoxystrobin, boscalid, clothianidin, fluroxypyr, glyphosate, imidacloprid, prothioconazole, thiamethoxam, thiacloprid, and the internal standards (IS), acetamiprid-d3, clothianidin-d3, imidacloprid-d4, thiacloprid-d4, thiamethoxam-d3, triphenyl phosphate (TPP), MCPA-d6 and malathion-d10 were purchased from Merck Life Science (Ireland). Deuterated MCPA was used as an internal standard for fluroxypyr, while malathion-d10 was used as an internal standard for azoxystrobin, boscalid and prothioconazole. The internal standards Glyphosate-^13^C_2_, ^15^N, and AMPA-^13^C, ^15^N were purchased from LGC standards (LGC, UK). Pesticide stock solutions were prepared in LC-MS grade acetonitrile, except for glyphosate and AMPA, which were prepared in deionised water [102]. Working standard solutions were diluted from stock solution before analysis. All working standard solutions were freshly prepared from stock on the analysis day and filtered through 0.45 μm pore-sized PTFE membrane filters before analysis. Silanised 2 mL amber autosampler vials were obtained from Agilent (Germany).

### 2.5 Soil pesticide extraction methods

The targeted pesticides, acetamiprid, AMPA, azoxystrobin, boscalid, clothianidin, fluroxypyr, glyphosate, imidacloprid, prothioconazole, thiacloprid, and thiamethoxam, were extracted using two different extraction methods, based on compound polarity. Glyphosate and AMPA were extracted using the modified QuPPe-PO method [103], while all the other analytes were extracted using the Dutch mini-Luke method [104]. The chemical properties of each of the pesticides are detailed in Table 1.

**Table 1:** Chemical properties of all the quantified pesticides [115,119,147,167,168].

Pesticides	Vp (mPa)	pKa	log *K*_*ow*_	*K*_oc_	DT_50_ (days)	WS (mg/L)	GUS index
Acetamiprid	1.73E-04	0.7	0.8	267	8.2	4250	0.94
Azoxystrobin	0.00E+00	NI	2.5	594	181	6	3.10
Boscalid	7.20E-04	NI	2.96	9500	578	4.6	2.66
Clothianidin	0.00E+00	11.9	0.905	60	1155	327	3.74
Fluroxypyr	4.00E-06	2.94	2.2	136	21	91	2.42
Imidacloprid	0.00E+00	1.56	0.57	800	190	610	3.69
Prothioconazole	4.00E-04	6.9	3.82	1765	1336	300	−0.18
Thiacloprid	0.00E+00	NI	1.26	1584	142	185	1.1
Thiamethoxam	7.00E-06	NI	−0.13	68.4	301	4100	3.58

Notation. Vp = vapour pressure; pKa = dissociation constant; NI = non-ionisable; log *K*_ow_ = partition coefficient; *K*_oc_ = soil adsorption coefficient; DT50 = half-life; WS = water solubility; GUS = Groundwater Ubiquity Score.

#### 2.5.1 Dutch mini-Luke method

An aliquot of 15 g air dried and sieved soil sample was weighed into a 250 mL PTFE centrifuge tube, and 15 mL deionised water was added and shaken vigorously for 1 min. Then, 30 mL of 1% acetic acid in acetone were added and homogenised using IKA Ultra-Turrax T-25 homogeniser (Ireland) at 1500 rpm for 30 s. Following the homogenising, 30 mL dichloromethane and 30 mL petroleum ether were transferred to the tube, and the sample mixture was homogenised again using the homogeniser at 1500 rpm for 30 s to induce phase separation. Then, the centrifuge tubes were centrifuged at 4000 rpm for 10 min, and 60 mL of the supernatant was carefully decanted into a 100 mL conical flask. The supernatant then evaporated under N_2_ gas flow to approximately 2 mL before being transferred to a 10 mL volumetric flask and made up to volume with ethyl acetate. Subsequently, 0.5 mL of the ethyl acetate sample was diluted into a 10 mL volumetric flask made up of volume with methanol. Finally, 1 mL aliquot of the methanol extract was filtered through a 0.22 μm hydrophilic PTFE syringe filter into a silanised autosampler vial for liquid chromatography-tandem mass spectrometry (LC-MS/MS) analysis.

#### 2.5.2 QuPPe-PO method

Two grams of homogenised air-dried soil samples were weighed into a 15 mL Falcon centrifuge tube. 2 mL of acidified deionised water (1% formic acid) was added and vortex mixing for 1 min, followed by 10 mL of acidified methanol (1% formic acid) addition and 1 min of vortex mixing. The centrifuge tube was then centrifuged at 4500 rpm for 10 min. Approximately 10 ml of supernatant was transferred into a silanised glass vial and concentrated to dryness under an N_2_ stream. The concentrated residue was re-dissolved in 1 mL of MeOH: H_2_O (50:50) solution before being filtered through a 0.22 μm hydrophilic PTFE syringe filter into a silanised autosampler vial for LC-MS/MS analysis.

### 2.6 LC-MS/MS methods and conditions

The pesticides, acetamiprid, azoxystrobin, boscalid, clothianidin, fluroxypyr, imidacloprid, prothioconazole, thiacloprid, and thiamethoxam, were analysed with the C18 column method. Meanwhile glyphosate and AMPA were analysed using a novel HILIC column method.Two multi-reaction monitoring (MRM) transitions were monitored for all the targeted compound, and the data acquisition are detailed in Table S2.

#### 2.6.1 C18 column method

LC-MS/MS analysis was performed using liquid chromatography (Agilent 1290 Infinity II) coupled with a triple quadrupole mass detector (Agilent 6470A) and XBridge UPLC BEH C-18 analytical column of 4.6 × 100 mm, 3.5 μm particle size (Waters Chromatography, Ireland). The sheath gas temperature was kept at 340 °C, and the sheath gas flow was 11 L/min 5 mM ammonium formate with 0.1% formic acid in deionised water (mobile phase A) and 0.1% formic acid in acetonitrile (mobile phase B) were used for the gradient program, which started at 30% B and increased to 50% in 5 min, was linearly increased to 100% B in 7 min, and it was held at 100% for 2 min before the column reconditioned back to 30% in 2 min. The flow rate was maintained at 0.5 mL/min, the column temperature was kept constant at 30 °C, and the injection volume was 10 μL.

#### 2.6.2 HILIC column method

Similar LC-MS/MS system used as above, and a Shodex HILICpak VT50-2D analytical column of 2 × 150 mm, 5 μm particle size. 10 mM ammonium bicarbonate in deionised water (mobile phase A) and pure acetonitrile (mobile phase B) were used for the gradient program. The flow rate was constant at 0.1 mL/min, with an injection volume of 30 μL. The gradient program started at 80% A and increased to 100% A in 5 min. The A% concentration was held for 8 min before the column was re-equilibrated back to 80% in 3 min. The sheath gas temperature was kept at 350 °C, and the sheath gas flow was 12 L/min.

### 2.7 Quality control

The extraction method and analytical performance validation were adapted from SANTE Guidance Document on analytical quality control and method validation procedures for pesticide residue analysis in food and feed [105]. Matrix-matched calibration standards for each targeted pesticide were prepared in a composite soil sample encompassing all the collected soil samples, with the linearity assessed using seven-point calibration curve, ranging between 0.05 μg/L to 100 μg/L (Fig. S1). Two types of IS were used, stable isotopically labelled IS for quantitative analysis, while TPP used quality control for extraction performance. The performance of the Dutch mini-Luke extraction method was evaluated using accuracy (recovery%) and precision (RSD%) studies. The sensitivity of the extraction method was assessed by determining the Limit of Detection (LOD) and Limit of Quantification (LOQ), calculated by taking into the slope of the calibration curve and the standard deviation of the slope, and multiplying by 3.3 and 10, respectively [105]. Recovery and precision assessment were measured by fortifying pesticide-free soil samples at two concentrations, 2.66 μg/kg, which is the standardised LOQ for all the pesticides, and five times the standardised LOQ, 13.3 μg/kg. Nearly all the targeted pesticides fulfilled the acceptance criteria of the validation parameters based on SANTE guidelines [105], where the average recovery was recommended to be in the range of 70–120%, with RSD% less or equal to 20% (Table 2), except for prothioconazole at LOQ level (61.7%), and both fortification levels for glyphosate (53.3% and 67.9%, respectively, and AMPA (59.8% and 68.7%, respectively). The unsatisfactory recovery% and RSD% can be explained based on the instrumental matrix effect (ME%) experienced by AMPA, glyphosate and prothioconazole (Table 2). ME% was calculated using the response of targeted pesticide response and the response of matching isotopically labelled IS, where 100% of the ME% value indicates no instrumental matrix effect. In comparison, values below 100% indicate a loss in response (ion suppression), and value above 100% indicates an increase in response (ion enhancement) [106]. Hence, based on the ME% value, AMPA, glyphosate and prothioconazole experience ion suppression from the soil matrix resulting in unsatisfactory recovery% and RSD%. However, as all targeted pesticides achieved good linearity and reproducibility of calibration curves (r^2^ > 0.890) and the response compensation with matching isotopically labelled IS, the quantitation analysis of this study is valid.

**Table 2:** Linearity, Recoveries%, RSD%, LOD, LOQ and ME% for all the target pesticides in the composite soil sample (number of replicates, *n* = 3)

No	Pesticides	Linearity (r^2^)	Fortification Levels (μg/kg)				LOD (μg/kg)	LOQ (μg/kg)	ME%
			2.66		13.3				
			Recovery%	RSD%	Recovery%	RSD%			
1	Acetamiprid	0.999	105.7	11	96.57	18.9	0.11	0.33	114.9
2	AMPA	0.890	59.8	44.9	68.7	31.1	1.74	5.28	71.5
3	Azoxystrobin	0.996	101.3	14.3	97.7	26	0.3	0.91	94.4
4	Boscalid	0.999	118.1	9.2	99.7	25	0.13	0.39	147
5	Clothianidin	0.999	118.6	3.8	105.7	5.6	0.07	0.23	86.6
6	Fluroxypyr	0.987	74.7	20.1	112	25.3	0.88	2.67	15.6
7	Glyphosate	0.943	53.3	47.1	67.9	27.6	1.03	3.11	58.8
8	Imidacloprid	0.999	108.6	15	96.8	4.6	0.09	0.27	81.6
9	Prothioconazole	0.998	61.7	31.4	95.6	25.9	0.44	1.32	79.2
10	Thiacloprid	0.999	115.6	9.3	98.8	11.7	0.08	0.24	80.7
11	Thiamethoxam	0.992	108.4	7.5	94.7	4.5	0.41	1.23	88.57

### 2.8 Statistical analysis

The data management, analysis and visualisation using three software; Microsoft Office Excel, R statistical software (version: 4.2.1) and Graphpad Prism (version: 9.4.1). Only pesticide concentrations quantified at or above LODs were used for data analysis. Soil samples with pesticide concentrations below LOQs but above LODs were recorded based on each method's individual pesticides' LODs to minimise bias of left-censored data [[107], [108], [109]]. Pearson correlation coefficients were used to study the relationship between concentration and frequency of soil pesticides detection to soil physicochemical properties and pesticide properties. The hierarchical cluster heatmap was determined using the *pheatmap* package in R, where it was performed using the log concentration of the quantified pesticides. The dendrogram of the heatmap influences the clustering pattern, where Euclidean distance metrics were used for complete clustering. *T*-tests were applied to compare the significance of log-transformed pesticide concentration fluctuation between the two-sampling time points (within 24 h and a week after pesticide application), with respect to the individual targeted pesticides.

## 3 Results and discussion

Pesticide compounds that reside within the soil can behave differently depending on their physicochemical properties, which include sorption coefficient, water solubility, and vapour pressure. In addition, their behaviour may be dictated by the physicochemical properties of the soil itself, such as soil pH, texture, organic matter, moisture, total nitrogen and phosphorus content [50]. Hence, for monitoring purposes, measuring and correlating the pesticide concentrations and physicochemical properties of both components is crucial to understanding the distribution observed here for a number of Irish agricultural soil.

### *3.1* *Soil physico*c*hemical properties*

Most soils studied here ranged from highly acidic to slightly basic, with pH_CaCl2_ values ranging between 3.3 and 7.5 (mean = 6) (Table 3). Meanwhile, the clay percentage ranged between 11 and 29% with a CoV of 27.62% (mean = 18%). Soil organic matter (%) ranged from 3.6% to 42.9%, with CoV values of 89.92%, indicating high degrees of uniformity. The mean value for % soil organic matter was 9.1 which is deemed to be high (i.e. > 5%; [110]), total nitrogen values (mg/kg) were more diversified, from 23.3 to 137.1 mg/kg (mean = 56.0 mg/kg, CoV = 47.1), compared to total phosphorus (mg/kg), which was more uniform across the sites with a CoV of 96.54% and a range of 1.5–52.6 mg/kg (mean = 11.0 mg/kg).

**Table 3:** Summary of the physicochemical properties for a selection of Irish soils (*n* = 25).

Soil Properties	min	Max.	Med	Me	SD	CoV	LQ.	UQ
%Clay	11	29	18	18	5	27.62%	14	21
%Silt	5	49	15	16	8	52.53%	12	19
%Sand	36	82	64	66	9	13.60%	62	71.5
Soil pH_CaCl2_	3.3	7.5	6.4	6.0	1.1	18.80%	5.1	7.1
Soil Organic Matter (%)	3.6	42.9	6.2	9.1	8.2	89.92%	5.7	8.7
Soil Moisture (%)	2.8	11.7	5.0	5.4	1.8	34.15%	4.3	5.8
Soil Water Holding Capacity (%)	5	31	9	12	7	54.37%	7	17
Total Phosphorus (mg/kg)	1.5	52.6	9.5	11.5	11.0	96.54%	5.4	12
Total Nitrogen (mg/kg)	23.3	137.1	54.3	56.0	26.4	47.1%	36.1	72.9

Notation. Min = minimum; Max = maximum; Med = median; Me = mean; SD = standard deviation; CoV = coefficient of variance; LQ = lower quartile; UQ = upper quartile.

### 3.2 Irish agricultural soil pesticide concentrations within 24 h of pesticide application

**Fig. 2:**
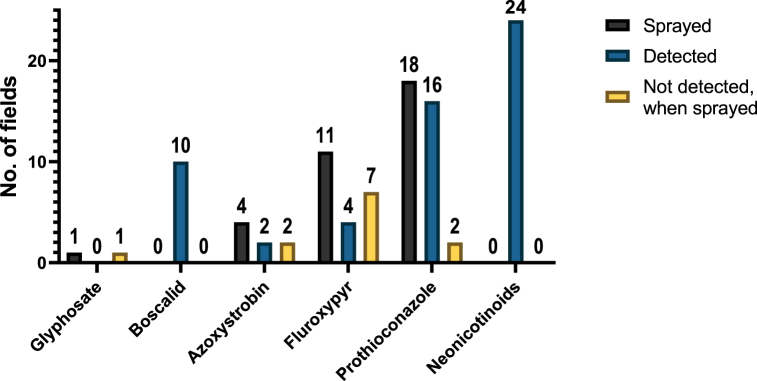
Applications and levels of detection for the different pesticide types investigated in this study.

Of the 25 sampling sites, 18 had been sprayed with prothioconazole, 11 with fluroxypyr, 4 with azoxystrobin, and 1 with glyphosate, with 14 sites having been sprayed with more than one targeted pesticide. No sites had been sprayed with boscalid or any of the five neonicotinoids. A summary of the nine pesticide residue concentrations detected at the 25 sampling sites is presented in Table 4. Overall, nearly all the targeted pesticides were detected, except for glyphosate and AMPA, even though glyphosate was applied in one of the 24 agricultural fields (Fig. 2) and has been detected in floral resources by Zioga et al. (2023), and both were removed from subsequent analyses. The total pesticide concentrations ranged from below the level of detection (n.d.) to 45.66 μg/kg; the highest concentration detected was the pesticide prothioconazole at 45.66 μg/kg. As for the detection rates, fluroxypyr is the least frequently detected, at 16% of all the sampled sites, while prothioconazole was detected at the rate of 64%. Interestingly, neonicotinoids were detected and quantified in the collected soil samples at detection rates ranging from 28 to 76%, with the maximum neonicotinoid concentration ranged between 0.178 μg/kg for acetamiprid to 1.466 μg/kg for thiamethoxam.

**Table 4:** Summary of the concentrations of analysed pesticides in Irish soils (*n* = 25).

Pesticides (μg/kg)	Min	Max	Me	Med	SD	CoV	Detection rates (all sites) (%)	Relative detection (Sites Detected: Sites Applied)
Acetamiprid	n.d.	<LOQ	<LOQ	<LOQ	–	–	28	7:0
Azoxystrobin	n.d.	2.523	0.823	0.283	0.933	113.4%	24	2:4
Boscalid	n.d.	2.72	1.09	0.987	0.847	77.75%	40	10:0
Clothianidin	n.d.	0.279	0.193	0.201	0.068	35.24%	32	8:0
Fluroxypyr	n.d.	<LOQ	<LOQ	<LOQ	–	–	16	4:11
Imidacloprid	n.d.	0.383	0.177	0.163	0.083	46.79%	36	9:0
Prothioconazole	n.d.	45.66	3.278	0.913	9.546	291.2%	64	16:18
Thiacloprid	n.d.	0.24	0.194	0.2	0.043	22.03%	76	19:0
Thiamethoxam	n.d.	1.466	1.349	1.429	0.195	14.43%	72	18:0

Notation. Min = minimum; Max = maximum; Me = mean; Med = median; SD = standard deviation; CoV = coefficient of variance; n.d. = not detected; and LOQ = Limit of Quantification.

Based on the spraying record, azoxystrobin, glyphosate, fluroxypyr, and prothioconazole were all applied within 24 h of sampling. However, in terms of detection, not all pesticides that were reported as being applied at the sampling site were detected in our analysis. As mentioned, glyphosate was not detected in the soil from the site where it was exclusively applied. Azoxystrobin, fluroxypyr and prothioconazole were detected in 50%, 36% and 89% of the sites where they had been reported to be applied, respectively. However, even though fluroxypyr were detected in four sites, all of them were found to be below the LOQ. Incidentally, prothioconazole was the most frequently and successfully detected pesticide in 16 of the 18 sites where it was applied, with only six sites above the LOQ. The fact that pesticides were not always detected in field soils where they were recently applied could be due to their failure to reach the soil within 24 h or reflect that the concentrations were below the detection limit in the soil samples collected. In addition, rapid biodegradation of the pesticides to CO_2_ and H_2_O or other degradation routes to partial metabolites may also result in a failure to detect them, also resulting in decreased toxicity [111,112].

As mentioned previously, Irish soils generally have a high organic matter percentage (Table 3), which could contain a more extensive microbial community [[63], [64], [65], [66]], resulting in rapid pesticide degradation. Even if pesticides were not degraded completely, partial degradation could result in higher mobility of the degraded product in the soil layers, a phenomenon observed for azoxystrobin and its more water-soluble degradation product [113].

Neonicotinoids were detected in 96% of the sampled fields, despite not being applied to all sites (Fig. 2). Our findings, although unexpected in the context of what compounds were applied, are consistent with the half-lives reported for neonicotinoids, i.e., that they are exceptionally long, and can differ significantly (31–450 days for acetamiprid, 148–7000 days for clothianidin, 28–1250 days for imidacloprid, 3.4–1000 days for thiacloprid and 7–335 days for thiamethoxam; which highlights the extent of their persistence in terrestrial environments [86,114]. Interestingly the fungicide boscalid was detected in the soils from ten sites, again without any report of recent application (i.e., this crop season). Unlike the neonicotinoids, boscalid is not banned for outdoor use; therefore, the detection of boscalid in soil samples could be due to the persistence of this compound from the previous crop season. The *K*_oc_ values for boscalid range from 507 to 1110 mL/g [115], suggesting that strong absorption into the soil, coupled with its low mobility, could result in significant persistence [116]. Such persistence is commonly reported, with an estimated 69% of pesticides detected in soils being attributed to applications from previous crop seasons [117] with some being reported up to 10 years after application [118]. The identification of non-recently applied pesticides could also be explained through drift processes from neighbouring other intensively managed agricultural fields (no data was obtained about land use or management in neighbouring sites).

**Fig. 3:**
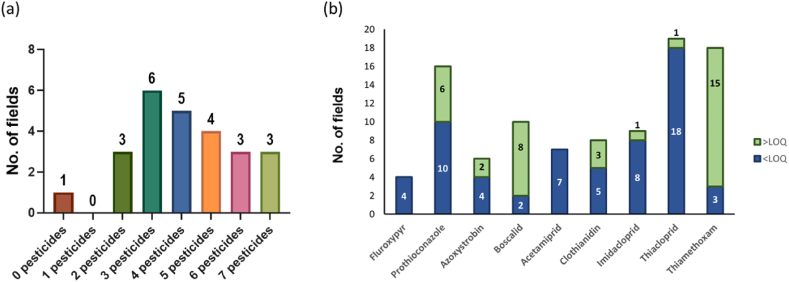
Distribution of quantified pesticides in Irish agricultural soils (a) studied fields based on the number of multi-residues (b) pesticide detection frequency and concentration.

Of the 25 sites included in this study, 23 sites had up to two target pesticide products applied in a single day application within 24 h of sampling. It was not unexpected therefore that 23 sites were determined to contain at least two or more pesticide residues (Fig. 3(a)). However, eighteen fields contained 2–5 residues, with six fields where a large number of residues (>6) were detected. Only one site had no detected pesticides, and no sites had just a single detectable pesticide. Taken together, these findings highlight that more than the recently applied pesticides are present in the soils sampled, which points to a pervasiveness of pesticides in Irish agricultural systems. These findings also indicate that, despite their ubiquitous detection, not all pesticides were present at high concentrations, and not all pesticides had similar accumulation profiles. For instance, both fluroxypyr and acetamiprid were detected in four and seven sites, respectively, but at concentrations below their LOQ values (Fig. 3 (b)), even though fluroxypyr had just been applied in 11 sites, and acetamiprid had not been applied in any. Another compound not applied in any site was thiamethoxam, but in contrast to acetamiprid, it was detected in 18 fields, with 15 fields above LOQ.

### 3.3 Identification of pesticides with land-use characteristics

**Fig. 4:**
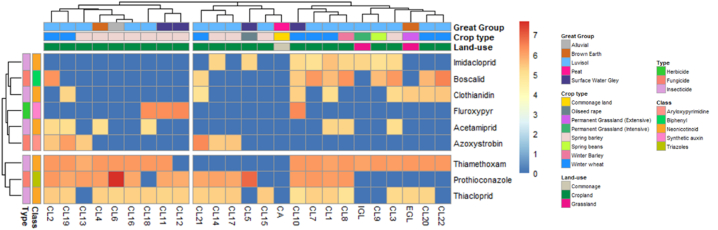
Hierarchical cluster heatmap analysis of nine detected pesticides clustered by concentration profiles of targeted pesticides and sampling site details. The colour of each cell represents the log_10_ pesticide concentrations. The dendrogram was cut to present two clusters of sample sites and two clusters of targeted pesticides. Notation. EGL = Extensive grassland; IGL = Intensive grassland; CL = Cropland; CA = Commonage area.

Hierarchical cluster analysis was used to visualise coherent patterns that emerged based on the concentration profiles of quantified pesticides expressed across the different pesticide types and soil classes (Fig. 4). Within the studied sites, we show for the first time that hierarchical clustering resulted in two distinctions of Irish agricultural soils, relating to low and high pesticides concentrations. The dendrogram illustrating the clustering of the pesticide classes and types (at the left border of Fig. 4) indicated that the cluster with the highest pesticide concentrations is dominated by thiamethoxam, prothioconazole and thiacloprid, which resulted in these pesticide groups being discriminated from other pesticides. In addition to higher pesticide concentrations, the cluster formation was also driven by the detection rate, where prothioconazole, thiamethoxam, and thiacloprid were detected in nearly all the sampled fields, with a detection rate of 64%, 72% and 76%, respectively (Table 3).

In addition, the top dendrogram (showing clustering based on the soil classes of the sites and pesticide concentrations) distinguished the two cluster. For instance, the cluster comprising low pesticide concentrations was associated with more diverse land-use types, including cropland, grassland and commonage land, whereas the cluster with high pesticide concentrations was associated with croplands only. Finally, luvisol and surface water gley soil types were largely associated with the high pesticide cluster, while the low pesticide cluster had a more heterogeneous mix of soil types.

Prothioconazole is one of the most widely used fungicides in Ireland and is applied to an estimated 5% of cultivated lands nationally [78]. Of the sites sampled in this study, 72% were reported to have received a recent application which explains its clustering with the other received a high concentration pesticide. Furthermore, other recently applied pesticides, such as fluroxypyr and azoxystrobin, were detected at a lower rate of 36% and 50% in the sprayed fields, respectively, compared to prothioconazole, which has a high detection rate of 89%, which could be due to the compound's chemical properties. Prothioconazole has an estimated *K*_oc_ value of 1765 [119], suggesting that this compound has low mobility in the soil layers. In addition, in acidic conditions prothioconazole has a log *K*_ow_ value of 4.16, indicating that it will readily be adsorbed to the organic matter component of the soil [120] justifying its failure to dissipate and its high detection levels in this study.

The quantification of high concentrations of neonicotinoids, specifically thiamethoxam and thiacloprid, relative to other recently applied pesticides is highly concerning. Our results indicate that the contamination of Irish agricultural soils with neonicotinoids is highly prevalent. Assuming that neonicotinoids had not been applied after their ban in 2018, their detection could be explained by their lack of degradation and movement through the environment. As soon as neonicotinoids come in contact with soil, multiple factors could influence their behaviour; the soil type, biotic and abiotic properties of the soil, and with the fluctuations of these factors, neonicotinoids can take multiple routes through the environment [121]. This type of mobility may explain the results observed in the extensive grassland (EGL) site where clothianidin, thiacloprid and thiamethoxam were detected. Historically, this site would not have been sprayed with neonicotinoids or planted with neonicotinoid treated seed, all of which were confirmed by the farmer; however, our findings clearly demonstrate the presence of neonicotinoids. Interestingly the commonage land (CA) site would also have had a history of no pesticide applications, but unlike EGL, no pesticides were detected in CA soil. Given that CA is a natural peatland (Fig. 4), which is unsuitable for agricultural cultivation; very little human activity has occurred on this land or in the surrounding areas. By contrasting these two sites with a similar pesticide application history, it can be determined that an agricultural field can be contaminated with pesticides even without direct application. There could also be multiple routes of contamination of EGL, such as run-off waters from adjacent fields, off-field dust, or spray-drift during treatment at a nearby field [25,[121], [122], [123]]. Similar findings were also reported by Ref. [124] where neonicotinoids, especially clothianidin, imidacloprid and thiacloprid, were detected in extensively managed agricultural land with no history of neonicotinoid application up to 10 years.

**Fig. 5:**
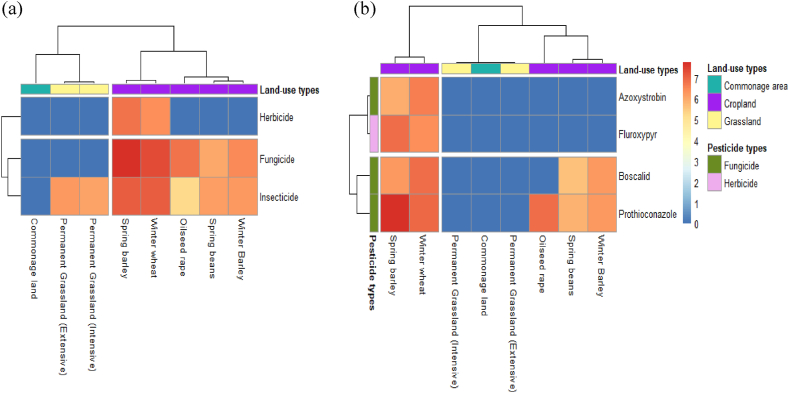
Simplified hierarchical cluster analysis heatmap clustered by log_10_ concentration profile (a) Crop types clustered by pesticide types (b) Crop types clustered by targeted pesticides (excluding neonicotinoid).

To simplify the heatmap the Irish soil great group and pesticide class variables were removed from the hierarchical clustering analysis, allowing us to focus on crop and pesticide types (Fig. 5(a)). Even without recent application, insecticides emerge as the most dominant pesticide type distinguished in the clustering as it is the only pesticide type detected in all agricultural fields, followed by fungicide and herbicide. For the land-use types, as the main driver of the clustering is pesticide concentration, and as higher concentrations were observed in the cropland sites, hierarchical clustering analysis yielded croplands clustering, while commonage land and grasslands formed a separate cluster. This clustering pattern also correlates with the recorded percentage of pesticide use in Ireland, where it has been reported that croplands are treated with 95.5% insecticides, 92.6% fungicides and 41% herbicides [78].

To further investigate the pesticides that are still used widely in an Irish context, hierarchical clustering was performed after removing neonicotinoids, basing the clustering on crop type and non-neonicotinoid pesticides (Fig. 5(b)). This had the result of collapsing the commonage land and grasslands into a single cluster with no targeted pesticides detected. The discrimination of spring barley and winter wheat from other crop types can be explained based on the cereal production area in Ireland. In 2021, of the 356.7 thousand hectares of area utilised for cereal production in Ireland, spring barley, winter barley, and winter wheat are the top three cereals grown, with 42%, 24% and 20% of the total area, respectively [125].

Additionally, when insecticides were removed from the analysis, it was observed that fungicides and herbicides were predominantly applied to croplands [78]. This is undoubtedly reflected in the hierarchical clustering where spring barley and winter wheat are grouped in the cluster for high pesticide concentrations, as these crop fields with all the targeted fungicides and herbicides at high concentrations (Fig. 5(b)). Notably, azoxystrobin is clustered with fluroxypyr, separated from other fungicides, while boscalid and prothioconazole cluster together as these pesticides are primarily detected in the croplands.

### *3.4* *Relationship between pesticides with soil physico*c*hemical and pesticide properties*

**Fig. 6:**
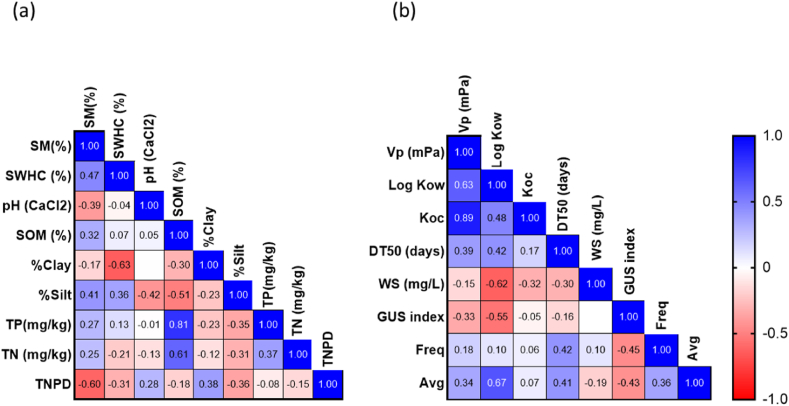
Pearson correlations coefficient plot based on the number of soils containing quantifiable pesticide residues (*n* = 24): (a) the total number of pesticide content is represented with the measured soil properties, (b) The frequency of pesticide detection and pesticide average concentrations correlated with their pesticide properties. Notation. SM = Soil Moisture; SWHC = Soil Water Holding Capacity; SOM = Soil Organic matter; TP = Total Phosphorus; TN = Total Nitrogen; TNPD = Total Number of Pesticides Detected; Vp = Vapour pressure; DT_50_ = Half-life; WS = Water Solubility; GUS = Groundwater Ubiquity Score; Freq = Frequency of detection; Avg = Average concentration “*N*”.

Pearson correlation analyses were carried out to determine the potential relationship between the soil physicochemical properties and the total number of pesticides detected quantified at the respective fields (Fig. 6(a)). Only one significant correlation was identified: a positive correlation between total number of pesticides detected and percentage of clay (p = 0.04), while weak correlations with the other measured soil physicochemical properties. A single statistical significance among 36 tests might be a statistical artefact, but a similar correlation has been reported by other studies, which suggests a substantial relationship. It is also noted that even though weakly correlated, total number of pesticides detected correlates negatively with soil organic matter.

This finding is contrary to many of the reported publications [2,[126], [127], [128], [129], [130]], where a positive correlation is generally observed for the relationship between pesticides and soil organic matter. However, this observation could be explained based on the Irish soil great group. Luvisol is a soil great group with a subsoil enriched with a higher percentage of clay, and as a more significant number of sampled sites were from the luvisol soil great group, clay particles could be the more dominant component that plays a role in the adsorption of pesticides. Even though the studied sites were also observed to have a high organic matter percentage (>5%), clay particles could be more readily available for pesticide adsorption. This postulation is supported by Durovic et al. (2009), where it was stated that even though soil organic matter and clay can both play a role in the adsorption of pesticides, in soils with a higher percentage of clay fractions, pesticide residues would readily adsorb clay component rather than soil organic matter [110]. Similar findings have also been reported in the Czech Republic, where pesticides were reported to correlate negatively with soil organic matter while correlating positively with clay minerals [131]. On the other hand, organic matter and the clay fraction could both exist in high concentrations, and the pesticides would still adsorb more readily to the clay particles. Theoretically, clay can physically coat and encapsulate soil organic matter, resulting in stable aggregate formation [58], protecting the organic matter from other soil properties, including the adsorption of pesticides. While the organic matter is rendered unavailable for pesticide adsorption due to encapsulation, the larger surface area of clay minerals', with –OH groups and transferable cations, can enhance the adsorption of pesticides [132]. Consequently, it increases pesticide compounds’ adsorption to the clay particle, resulting in a positive correlation of pesticide with clay percentage.

The average concentration of pesticides quantified was weakly correlated with their properties, such as vapour pressure, log *K*_ow_, *K*_oc_, DT_50_, water, solubility and Groundwater Ubiquity Score (GUS) index. In contrast, the average concentration of the pesticides had a significant positive correlation with log *K*_ow_ (p = 0.03) (Fig. 6(b)). This relation indicates that with increasing log *K*_ow_ values, the pesticide concentration in the soil increases, which conforms to widely reported studies [[132], [133], [134]].

Soil pH correlates positively with total number of pesticides detected. As the pH of the sampled sites was observed to be skewed towards an acidic soil environment (mean = 6.37) (Table 2), it could result in increased sorbing of pesticides and increased persistence [135]. Chemically, ionic and hydrophilic compounds were observed to efficiently bind to clay minerals [136], and many of the targeted pesticides are ionisable based on their pKa values (Table 1), confirming their high affinity of total number of pesticides detected towards the clay particles and increased persistence. Interestingly, it is observed in this study that even the non-polar and non-ionic compounds (log *K*_ow_ >1) (Table 1) were detected at high concentrations in soil layers with high clay percentage (Fig. 4). The findings suggest that even though the basic pesticide properties, such as pKa, log *K*_ow_, DT_50_, and solubility help predict its behaviour and persistence, these properties become less determinant in the real-world scenario where a more significant number of external factors are involved. The fact that most pesticide behaviour studies are conducted under laboratory conditions is an issue often mentioned in the literature [117], as are the difficulties in transferring this knowledge to highly complex and highly heterogenous soils found in real agricultural sites.

### 3.5 Assessment pesticide concentrations a week after application

Scrutinising the sites for further monitoring purposes, based on the detection and quantification of widely used pesticides, resulted in the selection of 13 sites out of the original 25 sites. Table 5 presents the summary of the pesticide concentrations, where the total pesticide concentrations within 24 h of pesticide application ranged from the level below the detection (n.d.) to 45.66 μg/kg, while the total pesticide concentrations after one week of pesticide application ranged from n.d. to 5.02 μg/kg. Even though the highest total pesticide concentrations decreased by 89%, in both sampling timepoints, the highest concentrations detected were of the pesticide prothioconazole. For the 13 sites, acetamiprid and fluroxypyr both remained under LOQ concentrations during both sampling timepoints, with detection rate increased by 31% for acetamiprid after one week, while there are no changes in detection rate for fluroxypyr.

**Table 5:** Summary of the concentrations of analysed pesticides in Irish soils within 24 h and one week after pesticide application (*n* = 13, that is, the number of sites where pesticides were recently applied).

Pesticides (μg/kg)	Sampling	Min	Max	Me	Med	SD	CoV	Detection rates	Relative detection (Sites Detected: Sites Applied)
								(13 sites) (%)	
Acetamiprid	24 h	n.d.	<LOQ	<LOQ	<LOQ	–	–	31	4:0
	One week	n.d.	<LOQ	<LOQ	<LOQ	–	–	62	8:0
Azoxystrobin	24 h	n.d.	2.523	0.319	1.323	0.757	80.51%	23	2:2
	One week	n.d.	2.071	0.159	1.03	0.574	0.00%	8	1:2
Boscalid	24 h	n.d.	1.59	0.142	0.795	0.437	117.90%	23	3:0
	One week	n.d.	1.034	0.099	0.517	0.284	82.02%	23	3:0
Clothianidin	24 h	n.d.	<LOQ	<LOQ	<LOQ	–	–	31	4:0
	One week	n.d.	0.271	0.044	0.136	0.094	11.63%	23	3:0
Fluroxypyr	24 h	n.d.	<LOQ	<LOQ	<LOQ	–	–	31	4:9
	One week	n.d.	<LOQ	<LOQ	<LOQ	–	–	31	2:9
Imidacloprid	24 h	n.d.	<LOQ	<LOQ	<LOQ	–	–	23	3:0
	One week	n.d.	–	–	–	–	–	0	0:0
Prothioconazole	24 h	n.d.	45.66	3.851	0.44	12.563	256.50%	85	11:11
	One week	n.d.	5.02	1.06	0.44	1.468	98.84%	85	11:11
Thiacloprid	24 h	n.d.	0.24	0.086	0.08	0.051	72.74%	92	12:0
	One week	0.194	0.242	0.234	0.238	0.013	5.58%	100	13:0
Thiamethoxam	24 h	n.d.	1.465	0.872	1.376	0.719	3.70%	62	8:0
	One week	n.d.	1.489	0.91	1.469	0.749	0.60%	62	8:0

Notation. Min = minimum; Max = maximum; Me = mean; Med = median; SD = standard deviation; CoV = coefficient of variance; n.d. = not detected; and LOQ = Limit of Quantification.

Comparing the detection rates of these widely used pesticides over one week indicates that, except for azoxystrobin, all the other pesticides were detected at the same rate. The detection rate of azoxystrobin decreased by 15% (three sites to one site), while the maximum concentration decreased by 18%, from 2.523 to 2.071 μg/kg. While the detection rate did not change for the other compounds, the maximum concentration decreased for boscalid and prothioconazole by 35% and 89%, respectively. Fluroxypyr was detected at the same rate (four sites) for both sampling timepoints, but after one week, it was detected in only two out of nine sites where it was recently applied, in addition to two where it was not.

The detection of neonicotinoids between the one-week sampling timepoint in Irish agricultural soils corresponds with their widely reported long half-lives [[83], [84], [85], [86], [87], [88]]. In contrast to other neonicotinoid analytes, imidacloprid is the only neonicotinoid that was not detected in the 13 sites after a week, while thiamethoxam was detected at the same detection rates (eight sites) between both sampling timepoints. Furthermore, in terms of total pesticide concentrations, nearly all the neonicotinoids were observed to increase in concentrations, with clothianidin, thiacloprid and thiamethoxam increased by 11%, 1%, and 4%, respectively. The increase in neonicotinoid concentrations, even though not applied recently, can be explained by the movement of these persisting analytes upon desorbing from the sites where they had been accumulated, either within the field or from neighbouring fields. This movement can be attributed to the long persistence of neonicotinoids in agricultural soils [124] and the high mobility of these analytes in the environment [[137], [138], [139]]. Hence, the detection of neonicotinoids during both sampling timepoints further strengthens the necessity for regular monitoring of neonicotinoids in the soil.

**Fig. 7:**
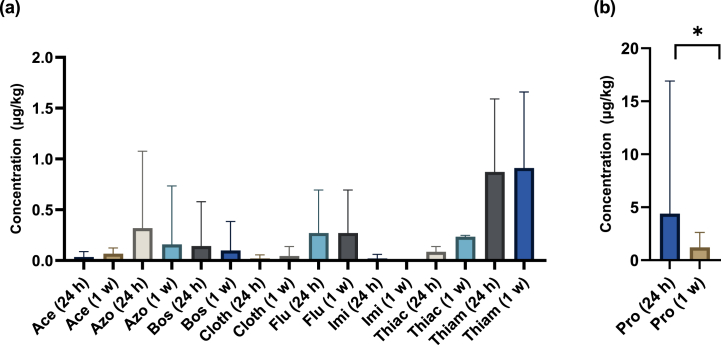
Fluctuation of the total concentration of individual pesticide analytes compared within 24 h and one week after pesticide application (*n* = 13), (a) all the targeted pesticides except prothioconazole, and (b) prothioconazole. Notation. Ace = Acetamiprid; Azo = Azoxystrobin; Bos = Boscalid; Cloth = Clothianidin; Flu = Fluroxypyr; Imi = Imidacloprid; Pro = Prothioconazole; Thiac = Thiacloprid; Thiam = Thiamethoxam; 24 h = within 24 h of pesticide application; and 1 w = after 1 week of pesticide application. The error bars represent SD.

Interestingly, even though the detection rates of most of the widely used pesticides were observed to be the same within 24 h and one week after pesticide application, the total pesticide concentration was observed to decrease between these two-sampling timepoints (Fig. 7). Prothioconazole were detected in all the fields that it was recently applied (11 sites) with the detection rate of 85%. However, prothioconazole is the only pesticide noted to decrease significantly (p = 0.03) in total concentration, with a decrease of 72% and only four of the 11 sites being above LOQ (Fig. 7(b)). Meanwhile, fluroxypyr were detected in four sites during both sampling timepoints, but with no concentrations above LOQ. Both azoxystrobin and boscalid concentrations decreased by 50% and 30%, respectively, with one site above LOQ for each analyte (Fig. 7(a)).

Overall, the dissipation of all the widely used pesticides between the two studied sampling timepoints can be attributed mainly to chemical or microbial degradation [140]. In this study, chemical degradation of the pesticides can be influenced by the clay and pH properties, highlighted previously as the only positively correlating soil physicochemical properties to the total pesticide detected in Irish agricultural soil. Similar findings have also been reported by Kah et al. (2007), where the author noted a positive correlation between the clay content and the degradation rate of pesticides [141]. The authors highlighted as the clay and organic matter content forms a significantly correlation, the degradation rate of pesticides could be catalysed by both of the soil components rather than just one of them [126]. This finding is also supported by Villaverde et al. (2008), where both clay and soil organic matter were noted to have a significant positive correlation with the degradation of dicamba, mesulfuron-methyl, 2,4-D, and flupyrsulfuron-methyl-sodium (p < 0.01) [142].

Based on the application history of the widely used pesticides, boscalid is the only pesticide that was not applied recently. Although previous studies had noted that boscalid tends to degrade slowly in soil, with half-lives ranging from 31.5 days to 180.1 days [143,144] up to 96–578 days [115]. Interestingly, in this study, the total concentration of this pesticide observed to decrease by 21% within the one week. The initial detection of boscalid in the 24-h soil samples, even when it is not applied, could be due to repeated application, as noted by Han et al. (2022), where degradation rates of boscalid decrease with frequency of treatment [143]. The author also noted that boscalid alters the soil microorganism structure with multiple applications resulting in the specialisation of pesticide-degrading species [143]. However, this shift in soil microbial structure could enhance the degradation rate of boscalid with time, which could be observed in this study. Similar findings have also been reported by Yu et al. (2009) with the application of fungicide carbendazim, where the author noted a 90% increase in degradation rate between the first and fourth fungicide application [145]. Nonetheless, even with a high biodegradation rate, due to the high persistence nature of boscalid, the repeated application of boscalid could result in substantial accumulated residues in soil, where its ecological effects in long-term contaminated soil remain uncertain.

Nevertheless, in the Irish agricultural soil, the decrease in total concentration of all the widely used pesticides can result from degradation catalysed primarily by microorganisms. The dominant role of soil microorganisms in the dissipation of pesticide residues in the soil is well known [53,146]. The postulation that microorganisms enhance the decreases is supported by the log *K*_OW_ values of azoxystrobin, boscalid, fluroxypyr and prothioconazole, 2.5, 2.96, 2.2 and 3.82 [119,141,147], indicating their great affinity to organic matter. As a higher percentage of soil microorganisms are housed in the soil organic matter phase, it increases the probability of contact between the adsorbed pesticides and microorganisms. When microorganisms come in contact with pesticides, the pesticides may be utilised as a source of carbon and energy, rapidly decreasing the pesticide concentrations [53]. This reasoning is supported by various studies [[148], [149], [150], [151]], where the alteration of organic matter in the soil increases microorganism activity, subsequently escalating pesticide degradation. The lower degradation rate of fluroxypyr compared to the other pesticides can also be linked to soil organic matter and microbial activity. Kah et al. (2007) had previously highlighted that the degradation rate of fluroxypyr correlates significantly (p < 0.05) to the soil organic carbon [141]. Thus, the lowest percentage of decrease of fluroxypyr can be recognised due to the lower organic matter content in Irish agricultural soil.

Despite the fact the pesticide prothioconazole decreases in total concentration significantly (p = 0.03), it does not necessarily mean the pesticide has dissipated entirely from the environment. It is highlighted by Lin et al. (2017), prothioconazole can rapidly degrade to its metabolite prothioconazole-desthio, with half-lives below 5.82 days, and the metabolite is found to persist longer in soils and plants compared to the parent compound [152]. Additionally, prothioconazole-desthio has a higher potency than prothioconazole due to its highly active state [153]. Hence, even though the concentration of the parent compound reduces rapidly, further studies are required to assess the potential unintended impact of the metabolite prothioconazole-desthio in the Irish agricultural soil layers.

### 3.6 Are the pesticide concentrations detected in Irish soils likely to pose an environmental risk?

The results of our study indicate that understanding pesticide contamination in Irish agricultural soils is complex - with pesticides applied in sites sometimes, but not always, being detected, and conversely, pesticides not applied being detected. An environmental risk assessment is beyond the scope of this study. Indeed, assessing pesticide risk in soils is difficult due to the lack of a maximum pesticide limit available for soils. It is known; however, that pesticides used in agriculture can pose a potential risk, by entering the water bodies via surface run-off or leaching [154], which would then threaten the drinking water resources. The Irish drinking water limit, which is also in agreement with the European drinking water regulation, stipulates individual concentrations of pesticides to be below 0.1 μg/L, while the parametric limit of total pesticide concentration is set to be below 0.5 μg/L [155], which is similar to the groundwater quality standard [156,157]. These limits are emphasised for treated drinking water, where the potential pesticides in surface waters and groundwaters are reduced during the water treatment.

Nonetheless, the complete removal of pesticides from natural water sources is complex, and the detection of pesticides in drinking waters has been reported on multiple occasions [[158], [159], [160], [161]]. Findings by Schipper et al. (2008) highlight that untreated drinking water can exceed the parametric limit set for the total pesticide (>0.1 μg/L) [162]. Even though it is difficult to establish the source of pesticide pollution in a water body, run-off and leaching of accumulated soil pesticides could be one of them [[163], [164], [165]].

**Fig. 8:**
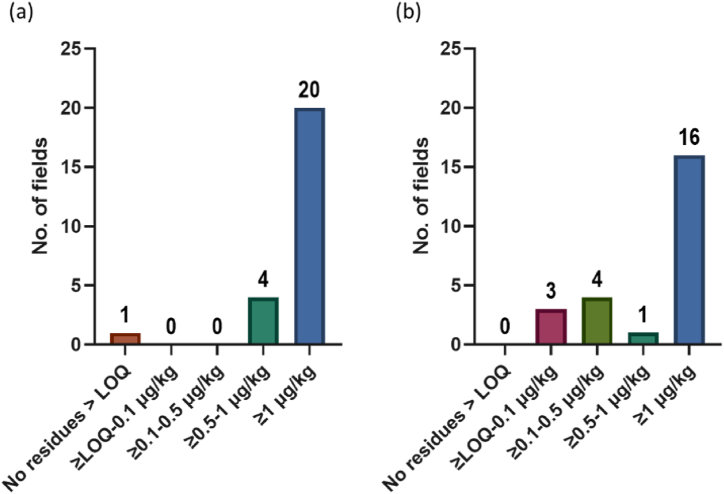
Pesticide concentrations at a field level, considering (a) total pesticide quantified and (b) pesticides that were detected but not applied (*n* = 25).

Considering pesticide concentrations at a field level, total pesticide concentrations, shown in Fig. 8 (a), indicate that 20 of the 25 sites have in excess of 1 μg/kg. In terms of the compounds applied within 24 h of sampling – prothioconazole, fluroxypyr, azoxystrobin – any potential risk could be interpreted as a “worst case scenario”, with very little time for degradation to occur; however, for the compounds detected that were not recently applied, namely boscalid and the neonicotinoids – this scenario consideration does not apply. As a result therefore, simply considering the total pesticide concentrations is not entirely appropriate, given that some compounds were applied within 24 h of sampling. When these compounds were removed from calculations, such that the only compounds considered were those that were not applied, 16 sites still had pesticide concentrations in excess of 1 μg/kg.

**Fig. 9:**
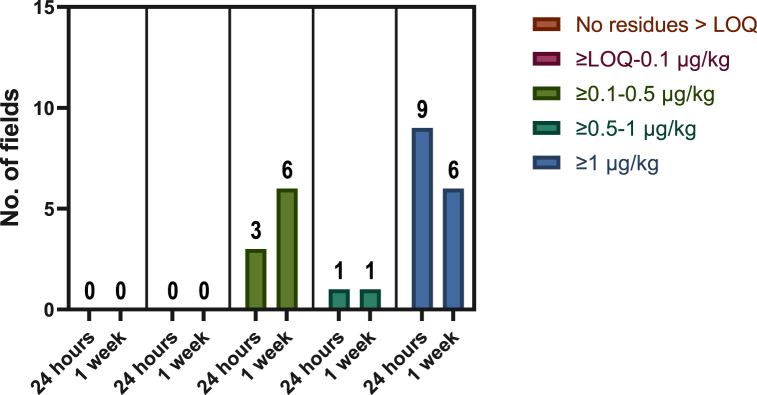
Field level total pesticide concentration of widely used pesticides within 24 h and one week after pesticide application (*n* = 13).

Each of the 13 sites that were found to have widely used pesticide concentrations when sampled within 24 h of application were also sampled 1 week post application. There was an increased number of fields with concentrations of ≥0.1–0.5 μg/kg, while the number of sites between the concentrations of ≥0.5–1 μg/kg remained the same for both sampling timepoints (one site), while sites with total concentrations higher than 1 μg/kg decreased from nine to six sites (Fig. 9). It can be inferred that the increasing number of fields in other lower pesticide concentration threshold categories is due to the decreasing total concentrations of the higher pesticide threshold categories. As highlighted before, boscalid and prothioconazole were identified to have a higher risk of persistence based on the physicochemical properties of both the soil and the pesticide. However, we noted that both boscalid and prothioconazole decrease in concentration, with 30% and 72%, respectively (Fig. 7). This highlights the difficulty of predicting the behaviour of pesticide compounds in the environment, strengthening the need for continuous pesticide monitoring in the soil.

### 3.7 Factors impacting the extent to which pesticide compounds can leach from soil to groundwater

The risk of the pesticides leaching through the soil layers and contaminating groundwater can be predicted by other physicochemical characteristics, particularly the percentage of sand content. In this study, an average of 65.6% of the sampled sites were deemed to have high sand content (>45%). Although the soil layers are noted to have high sand content, which usually coincides with high levels of leaching [166], our detection of multiple pesticides at significant quantities and in addition to the high clay content, allows us to conclude that leaching levels are low for the sites included in this study.

Conversely, the risk of pesticide transmission reduces significantly with increasing clay percentage with McGinley et al. (2022), reporting that soils with <20% clay have the highest risk of pesticide leaching through their layers and contaminating ground waters [166]. Considering that the mean clay percentage in the sampled sites is 18.4%, it can be established that pesticide movement in the Irish agricultural soil is very low. This statement is further supported by ElGouzi et al. (2012), in their study of phenylurea pesticide adsorption potentials, where pesticide leaching decreases and retention increases with increasing clay content [169]. The risk assessment can be further supplemented by considering the pesticide properties. Adsorption coefficients (*K*_oc_ and log *K*_ow_) can be used to predict the leaching potential, where the smaller the *K*_oc_ value, the more mobile a pesticide compound would be, while the more hydrophilic a compound is (log *K*_ow_) higher the leaching potential. Hence, by linking with the water solubility and GUS index values, the leaching potential of individual pesticides can be isolated.

Based on these pesticide properties, acetamiprid (not applied) and azoxystrobin (applied within 24 h) were identified as the pesticides with a higher risk of leaching relative to the other pesticides. Even though, both of these compounds have low log *K*_ow_ and *K*_oc_ values, with high-water solubility, the GUS value of 0.94 for acetamiprid indicates that this compound has a very low leaching potential [121], however, azoxystrobin identified to have a very high leaching potential with GUS value of 3.10 [147] (Table 1). On the other hand, boscalid (not applied) and prothioconazole (applied within 24 h) were identified to have a higher probability of persisting in the soil rather than leaching due to their high affinity towards the soil and high hydrophobicity.

The pesticide concentrations detected (Table 3) may also be used to assess the potential risks of individual pesticides. For example, acetamiprid was detected at levels of 0.1 μg/kg, with a maximum concentration of 0.178 μg/kg, indicating a lower risk of persistence. By contrast, azoxystrobin, with a concentration of 2.523 μg/kg could be considered as a risk to potentially contaminate groundwater [155]. This assessment of potential contamination is supported by McGinley et al. (2022), who scored the compound 27 on a transmission risk ranking scale of 9–42 [166]. Again, it should be considered that this concentration was detected within 24 h of application. Even though other targeted pesticides have some leaching potential, not all their properties lead towards it. For instance, clothianidin and imidacloprid both have high water solubility and log *K*_ow_ values; however, the GUS index of 3.74 and 3.69, respectively, indicates that these pesticide compounds have a very low leaching potential. In summary, therefore, the higher leaching potential risk was predictive for only two of the nine quantified pesticides, indicating a higher risk of pesticide accumulation than leaching. In relation to neonicotinoids in particular, acetamiprid has a higher risk of leaching, whereas clothianidin and imidacloprid have higher risks of accumulating. In this study, acetamiprid was the neonicotinoid detected least frequently, and the only one never to exceed the LOD. However, as we have no historical application information, it is not possible to evaluate if this relative detection pattern is related to extent of leaching or accumulation.

Even though the accumulation of pesticides in the soil layers reduces the transport of those compounds through the environment, this does not automatically lead to the conclusion that they do not pose a potential hazard to human health. For instance, high concentrations of the accumulated pesticides would have a lower degradation rate, as highlighted by Fogg et al. (2003) [170]. The authors reported in their study on biobeds that the rate of degradation decreases with increasing concentrations of the pesticides isoproturon and chlorothalonil. If the availability of pesticides increases in the soil, consequently, it would be expected to lead to biological uptake and bioaccumulation. Wang et al. (2021) noted that acetamiprid, imidacloprid and azoxystrobin can be translocated from soil to maize leading to bioaccumulation based on their bioavailability [171]. The authors also stated that pesticides with higher log *K*_ow_ value have a higher risk of accumulating in maize's roots, and pesticides with lower log *K*_ow_ have a higher risk of translocating to the shoots from the roots. Similar findings were reported by Li et al. (2018), where out of five neonicotinoids studied, acetamiprid was identified to have a greater risk of accumulating in the Japanese mustard spinach vegetable shoots, while thiamethoxam was noted to accumulate in the vegetable roots [172]. Ultimately, it needs to be considered that both leaching and accumulation potentially pose a risk of indirectly increasing the risk to food and environmental safety.

## 4 Conclusion

This study investigated the levels of pesticide contamination in Irish agricultural soils. Soil samples were collected from 25 sites: 22 croplands, two grasslands and one commonage land. Pesticides were applied to 23 of these sites within 24 h of sampling. From these fields, it was determined that 96% of the soil samples had detectable pesticide concentrations, where the concentrations of pesticides detected ranged from 0.18 to 45.7 μg/kg. Even though there was no recent application history at any of the sites, neonicotinoids were detected in 24 agricultural fields, even in a permanent grassland with no history of pesticide use. Similarly, to neonicotinoids, boscalid was detected in 10 sites even though it was not applied recently. However, glyphosate or AMPA were not detected in the single site where glyphosate was recently applied. Based on the distribution of quantified pesticide residues, 21 sites were shown to have three or more pesticide residues, with three fields having seven residues, against a baseline of no more than 2 pesticides applied recently at any individual site. Of the pesticides that were applied to the fields sampled, prothioconazole was the most detected pesticide, with the highest concentration quantified is 45.7 μg/kg and a detection rate of 89% in the sites where it was reported to be applied. Based on the hierarchical clustering analysis, luvisol is identified as the soil great group that dominates the highest pesticide concentrations cluster, croplands discriminate themselves from other land-use types, and spring barley and winter wheat are the crop types of fields that have the highest concentration of fungicides and herbicides. Notably, through Pearson correlation analysis, it was found that the total pesticide detected, and clay fraction forms a significant positive correlation with the total pesticide detected (p < 0.05). At the same time, log *K*_ow_ correlated significantly (p < 0.05) with total pesticide concentrations. 23 sites were shown to have total pesticide concentrations that exceeded 0.5 μg/L. When recently applied compounds were removed from calculations, 17 sites still had pesticide concentrations above 0.5 μg/L. Considering both the sampled agricultural soil properties and the individual pesticide properties, acetamiprid and azoxystrobin were identified to have higher leaching potential, while boscalid and prothioconazole to have accumulation risk. However, further monitoring of the sites where widely used pesticides were detected, revealed that all the widely used pesticides decreased in concentrations, in the range of 18–72%, with highest decrease observed in the case of prothioconazole. Even though boscalid was identified to have higher persistence risk, within the one-week timepoint, the pesticide is observed to decrease by 30%. Two main findings emerged from this study: firstly, in 15 of 25 sites analysed, pesticides not applied recently were detected in the soils sampled. Secondly, where pesticides were applied recently, this did not automatically result in quantifiable concentrations of pesticides in the corresponding soils. Fluroxypyr was only detected in 4 of 11 sites where it was applied, and never above the LOQ. Whilst prothioconazole was detected more frequently, in 16 of 18 sites where it was applied, it was below the LOQ in 10 of these sites. Correlations were observed between pesticide concentrations and the physiochemical properties of both the pesticides and soils. Future studies are needed to expand the number of pesticide compounds investigated, to evaluate the extent to which applied pesticides degrade, leach or accumulate in soils, and the factors that impact this, and to determine the resultant potential risks of pesticide soil contamination on the Irish environment.

## Author contribution statement

Mathavan Vickneswaran: Conceived and designed the experiments; Performed the experiments; Analysed and interpreted the data; Contributed reagents, materials, analysis tools or data; Wrote the paper.>

James C Carolan; Matthew Saunders; Blanaid White: Conceived and designed the experiments; Analysed and interpreted the data; Contributed reagents, materials, analysis tools or data; Wrote the paper.>

## Data availability statement

Data included in article/supplementary material/referenced in article.

## Declaration of competing interest

The authors declare the following financial interests/personal relationships which may be considered as potential competing interests:
