# Supplementary material for: Establishing the extent of pesticide contamination in Irish agricultural soils
Source: Heliyon. 2023 Aug 23;9(9):e19416. doi: 10.1016/j.heliyon.2023.e19416 (PMC10478240; doi:10.1016/j.heliyon.2023.e19416)
Supplement: Multimedia component 1 [file mmc1.docx]

**Supplementary document**

**Table S1** Details of the sampled sites and the pesticide applied

| **Field label** | **Cropland Type** | **Pesticide application record** | **Concentration applied (l/ha)** | **Field size (ha)** |
| --- | --- | --- | --- | --- |
|  |  |  |  |  |
| EGL | Permanent Grassland | No history of pesticide application | - | 5.3 |
| IGL | Permanent Grassland | Glyphosate | 4 | 7 |
| CL1 | Winter Wheat | Prothioconazole | 0.6 | 5 |
| CL2 | Winter Wheat | Fluroxypyr | 1 | 7 |
|  |  | Prothioconazole | 0.5 |  |
| CL3 | Spring Barley | Fluroxypyr | 1 | 6 |
|  |  | Prothioconazole | 0.5 |  |
| CL4 | Spring Barley | Fluroxypyr | 0.5 | 8 |
|  |  | Prothioconazole | 0.7 |  |
| CL5 | Oilseed rape | No recent application of targeted pesticides | - | 9.3 |
| CL6 | Spring Barley | Fluroxypyr | 0.5 | 8 |
|  |  | Prothioconazole | 0.25 |  |
| CL7 | Winter Wheat | No recent application of targeted pesticides | - | 4.2 |
| CL8 | Winter Barley | Prothioconazole | 0.8 | 4.45 |
| CL9 | Spring Bean | No recent application of targeted pesticides | - | 7.7 |
| CL10 | Winter Wheat | Fluroxypyr | 0.77 | 8 |
| CL11 | Spring Barley | Fluroxypyr | 0.77 | 6.4 |
|  |  | Prothioconazole | 0.46 |  |
| CL12 | Spring Barley | Fluroxypyr | 0.77 | 6.4 |
|  |  | Prothioconazole | 0.46 |  |
| CL13 | Spring Barley | Prothioconazole | 0.7 | 20 |
| CL14 | Spring Barley | Prothioconazole | 0.7 | 8 |
| CL15 | Spring Barley | Fluroxypyr | 0.6 | 1 |
|  |  | Prothioconazole | 0.5 |  |
| CL16 | Spring Barley | Fluroxypyr | 0.6 | 1 |
|  |  | Prothioconazole | 0.5 |  |
| CL17 | Spring Barley | Fluroxypyr | 0.6 | 1 |
|  |  | Prothioconazole | 0.5 |  |
| CL18 | Spring Barley | Fluroxypyr | 0.6 | 1 |
|  |  | Prothioconazole | 0.5 |  |
| CL19 | Winter Wheat | Azoxystrobin | 0.5 | 11 |
|  |  | Prothioconazole | 0.5 |  |
| CL20 | Winter Wheat | Azoxystrobin | 0.5 | 7.6 |
|  |  | Prothioconazole | 0.5 |  |
| CL21 | Winter Wheat | Azoxystrobin | 0.5 | 5 |
|  |  | Prothioconazole | 0.5 |  |
| CL22 | Winter Wheat | Azoxystrobin | 0.5 | 0.9 |
|  |  | Prothioconazole | 0.5 |  |
| CA | Commonage area | No history of pesticide application | - | - |

**Table S2** Acquisition and chromatographic parameters for the targeted pesticides

| Number | Pesticide | T_R_ (min) | MRM 1 | CE 1 | MRM 2 | CE 2 | Polarity |
| --- | --- | --- | --- | --- | --- | --- | --- |
| 1 | Acetamiprid | 4.39 | 223.2 > 126.1 | 20 | 223.2 > 56.1 | 20 | + |
| 2 | AMPA | 5.71 | 110.0 > 63.0 | 20 | 110.0 > 79.0 | 36 | - |
| 3 | Azoxystrobin | 9.66 | 404.0 > 372.0 | 19 | 404.0 > 344.0 | 27 | + |
| 4 | Boscalid | 9.89 | 343.0 > 307.0 | 20 | 343.0 > 272.0 | 32 | + |
| 5 | Clothianidin | 3.82 | 250.0 > 169.0 | 12 | 250.0 > 132.0 | 12 | + |
| 6 | Fluroxypyr | 5.90 | 255.0 > 181.0 | 24 | 255.0 > 209.0 | 12 | + |
| 7 | Glyphosate | 12.56 | 168.0 > 63.0 | 32 | 168.0 > 150.0 | 8 | - |
| 8 | Imidacloprid | 4.14 | 256.2 > 175.2 | 25 | 256.2 > 209.0 | 20 | + |
| 9 | Prothioconazole | 10.52 | 344.0 > 326.0 | 8 | 346.0 > 328.0 | 20 | + |
| 10 | Thiacloprid | 5.37 | 253.0 > 126.0 | 36 | 253.0 > 90.0 | 50 | + |
| 11 | Thiamethoxam | 3.27 | 292.0 > 211.1 | 15 | 292.0 > 181.0 | 24 | + |

**(f)**

**(e)**

**(d)**

**(c)**

**(b)**

**(a)**

**(k)**

**(j)**

**(i)**

**(h)**

**(g)**

**Fig. S1.** Calibration curve, R^2^, and the range of all the targeted pesticides: (a) acetamiprid, (b) AMPA, (c) azoxystrobin, (d) boscalid, (e) clothianidin, (f) fluroxypyr, (g) glyphosate, (h) imidacloprid, (i) prothioconazole, (j) thiacloprid, and (k) thiamethoxam.


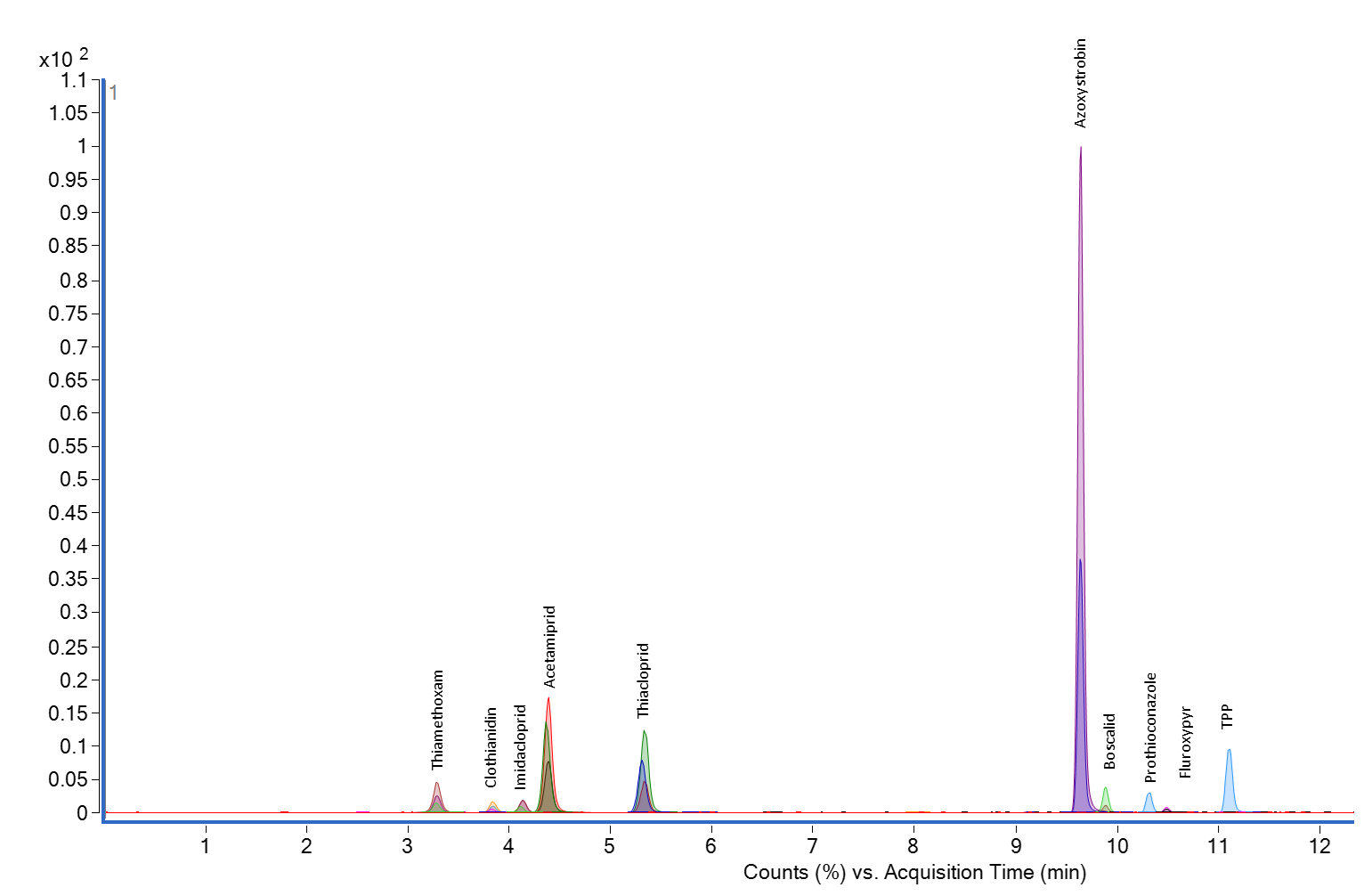

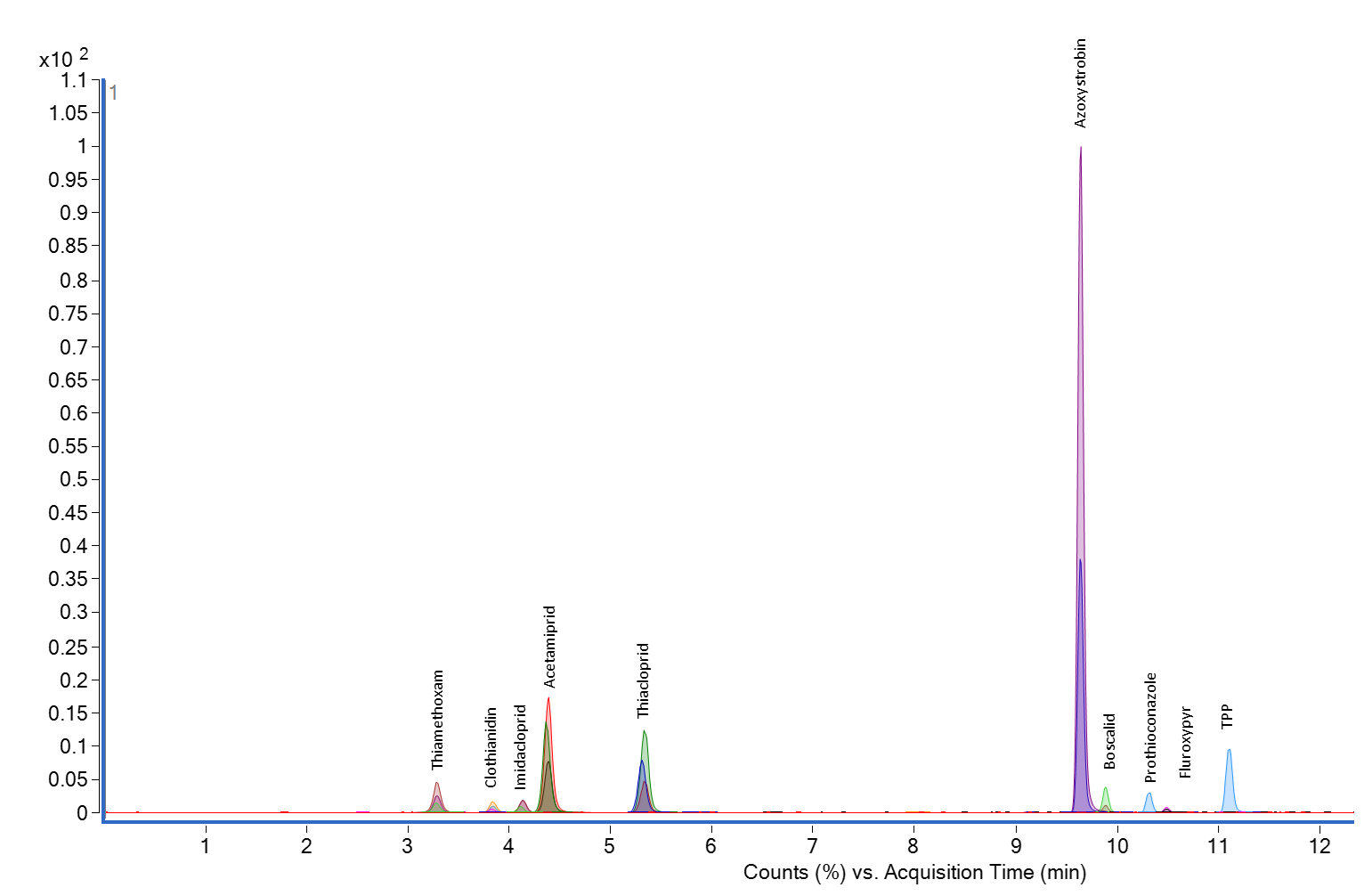


**Fig. S2.** Sample of the targeted pesticides standard’s chromatogram, injection concentration of 50 µg/kg.
